# An Effective Sodium-Dependent Glucose Transporter 2 Inhibition, Canagliflozin, Prevents Development of Hypertensive Heart Failure in Dahl Salt-Sensitive Rats

**DOI:** 10.3389/fphar.2022.856386

**Published:** 2022-03-09

**Authors:** Lili He, Sai Ma, Qingjuan Zuo, Guorui Zhang, Zhongli Wang, Tingting Zhang, Jianlong Zhai, Yifang Guo

**Affiliations:** ^1^ Department of Internal Medicine, Hebei Medical University, Shijiazhuang, China; ^2^ Department of Geriatric Cardiology, Hebei General Hospital, Shijiazhuang, China; ^3^ Department of Internal Medicine, Hebei General Hospital, Shijiazhuang, China; ^4^ Department of Cardiology, The Third Hospital of Shijiazhuang City Affiliated to Hebei Medical University, Shijiazhuang, China; ^5^ Department of Physical Examination Center, Hebei General Hospital, Shijiazhuang, China; ^6^ Department of Cardiology, Hebei General Hospital, Shijiazhuang, China

**Keywords:** heart failure with preserved ejection fraction, hypertension, myocardial hypertrophy, canagliflozin, fibrosis, metabolism

## Abstract

**Background:** The aim of the study was to investigate the protective effect of canagliflozin (CANA) on myocardial metabolism and heart under stress overload and to further explore its possible molecular mechanism.

**Methods:** High-salt diet was used to induce heart failure with preserved ejection fraction (HFpEF), and then, the physical and physiological indicators were measured. The cardiac function was evaluated by echocardiography and related indicators. Masson trichrome staining, wheat germ agglutinin, and immunohistochemical staining were conducted for histology analysis. Meanwhile, oxidative stress and cardiac ATP production were also determined. PCR and Western blotting were used for quantitative detection of related genes and proteins. Comprehensive metabolomics and proteomics were employed for metabolic analysis and protein expression analysis.

**Results:** In this study, CANA showed diuretic, hypotensive, weight loss, and increased intake of food and water. Dahl salt-sensitive (DSS) rats fed with a diet containing 8% NaCl AIN-76A developed left ventricular remodeling and diastolic dysfunction caused by hypertension. After CANA treatment, cardiac hypertrophy and fibrosis were reduced, and the left ventricular diastolic function was improved. Metabolomics and proteomics data confirmed that CANA reduced myocardial glucose metabolism and increased fatty acid metabolism and ketogenesis in DSS rats, normalizing myocardial metabolism and reducing the myocardial oxidative stress. Mechanistically, CANA upregulated p-adenosine 5′-monophosphate-activated protein kinase (p-AMPK) and sirtuin 1 (SIRT1) and significantly induced the expression of peroxisome proliferator-activated receptor gamma coactivator-1 alpha (PGC-1a).

**Conclusion:** CANA can improve myocardial hypertrophy, fibrosis, and left ventricular diastolic dysfunction induced by hypertension in DSS rats, possibly through the activation of the AMPK/SIRT1/PGC-1a pathway to regulate energy metabolism and oxidative stress.

## Introduction Context

Worldwide, 1–2% of the general adult population suffers from heart failure (HF), which comes with reduced quality of life, high morbidity, high mortality, and significant economic costs (Metra et al., 2017). According to systolic function, there are three types of HF: heart failure with reduced ejection fraction (HFrEF), heart failure with mid-range ejection fraction (HFmrEF), and heart failure with preserved ejection fraction (HFpEF) (Lytvyn et al., 2017). Among them, HFpEF is considered a precursor of HF in asymptomatic patients (Dunlay et al., 2017), and patients with HFpEF often have a higher burden of comorbidities, such as hypertension, obesity, diabetes, and chronic kidney disease (Lam et al., 2018). Currently, despite advances in drug therapy for HF, patients are still at increased risk of morbidity and mortality. Also, these treatments may bring serious side effects, including hypotension, kidney dysfunction, and electrolyte abnormalities (Abdalla et al., 2001). Therefore, identifying novel therapeutic strategies to improve symptoms, reduce mortality, and reduce acute decompensation is critical for the progression of HF outcomes.

Sodium-glucose co-transporter 2 inhibitors (SGLT2is) are novel hypoglycemic drugs that simulate fasting by promoting the secretion of urine sugar and increasing the levels of fatty acids and ketones (Mudaliar et al., 2016; Mcmurray et al., 2019). Multiple clinical studies have shown that the SGLT2i can significantly reduce the incidence of primary composite endpoint events (cardiovascular death and hospitalization due to heart failure) and can improve chronic HF (regardless of EF and with or without diabetes) (Perkovic et al., 2019; Anker et al., 2020; Packer et al., 2021). Among them, canagliflozin (CANA) is the first SGLT2i approved by the US Food and Drug Administration (Haas et al., 2014), but its benefit mechanism for HF patients remains unclear. A previous study has shown that empagliflozin can improve cardiac function, myocardial hypertrophy, and fibrosis in non-diabetic rats with left ventricular (LV) dysfunction after myocardial infarction and has been associated with normalization of myocardial glucose and fatty acid oxidative metabolism and increased ketogenesis (Yurista et al., 2019). Similarly, in a pig model of non-diabetic HF, empagliflozin also increased the consumption of cardiomyone bodies, reduced cardiac glucose utilization, and improved LVEF (Santos-Gallego et al., 2019). In addition, it is noteworthy that CANA can also induce transcriptional reprogramming to activate catabolic pathways and increase fatty acid oxidation and ketogenesis (Osataphan et al., 2019). Although regulation of myocardial metabolism by the SGLT2i has been shown to alter these beneficial effects in models of HFrEF, whether the same pathway exists in hypertension-induced HFpEF and the exact regulatory mechanisms remain unknown.

Inhibition of SGLT2 promotes urine sugar excretion and triggers a fast-like transcription pattern. In response to caloric restriction, the liver sirtuin 1 (SIRT1)/peroxisome proliferator-activated receptor gamma coactivator-1 alpha (PGC-1a) pathway is activated to maintain blood glucose, which leads to many changes in glucose metabolism and maintains the blood glucose at narrow levels (Rodgers et al., 2005). It was reported that knockdown of SIRT1 in mice caused mild hypoglycemia when fasting, increased systemic glucose and insulin sensitivity, and reduced glucose production (Rodgers and Puigserver, 2007). Similarly, caloric restriction also activated the SIRT1/adenosine 5′-monophosphate-activated protein kinase (AMPK)/PGC-1a pathway in myocardium, regardless of the SGLT2 expression (Ma et al., 2020). Based on the previously mentioned background, we hypothesized that SIRT1 played a key regulatory role in the aforementioned effects caused by SGLT2.

Hence, in this study, we investigated the effects of CANA on myocardial metabolism and cardiac function in Dahl salt-sensitive (DSS) rats with hypertension induced by 8% salt diet and further explored the possible mechanism of CANA acting on HF.

## Materials and Methods

### Animals and Groups

A total of 28 specific pathogen-free male DSS rats weighting 200.0–270.0 g (age, 7 weeks old) were purchased from Beijing Vital River Laboratory Animal Technology Co., Ltd. They were housed in an animal barrier system at the Clinical Research Center of Hebei General Hospital, with the room temperature controlled at 23–25°C and relative humidity about 60% in a 12-h light/dark cycle. After 1 week of adaptive feeding (0.3% NaCl AIN-76A), the DSS rats were randomly assigned to a high-salt diet (HSD, AIN-76A + 8% NaCl with irradiation) to induce HFpEF (*n* = 8), or to a normal-salt diet (NSD, AIN-76A + 0.3% NaCl with irradiation) to serve as controls (*n* = 10), or to a high-salt diet combined with 20 mg/kg/d CANA (HSD + CANA) to serve as the CANA intervention group (*n* = 10). DSS rats in the HSD group and NSD group received only the vehicle 0.5% hydroxypropyl methylcellulose at a volume of 5 ml/kg, orally and daily for 12 weeks. The animal experiment was approved by the Animal Ethics Committee of Hebei General Hospital and complied with the International Laboratory Animal Management Regulations.

### Blood Pressure Measurement

After 1 week of adaptive feeding, the tail blood pressure of DSS rats was monitored by using a tail sphygmomanometer (BP-2000, Visitech Systems, Inc., United States). Blood pressure was measured once a week, and six effective blood pressure values were taken to calculate the average value.

### Plasma and Urine Collection

One week before the end of the experiment, the urine of the rats in the metabolic cage within 24 h was collected under the condition of feeding and drinking. After 12 weeks of high-salt feeding, the rats were let to fast overnight, and blood was collected on an empty stomach. First, the rats were anesthetized with 3% sodium pentobarbital (30 mg/kg) by intraperitoneal injection. Then, whole blood was collected from the abdominal aorta, and then, the serum was collected and stored at −80°C. An automatic biochemical analyzer (DS-261, SINNOWA Medical Science and Technology Co., Ltd., China) was used to detect serum albumin, serum sodium, serum creatinine, total cholesterol, low-density lipoprotein cholesterol (LDLC), triglycerides, 3-hydroxybutyric acid, and urine glucose, urine sodium, urine albumin, and urine creatinine. A rat BNP ELISA kit (CSB-E07972r, CUSABIO, China) was used to measure circulating hormones.

### Oral Glucose Tolerance Test and Insulin Level Measurement

The OGTT was performed on the 12th week of the high-salt diet intervention. Briefly, six rats in each group were randomly selected, and glucose was given orally after overnight fasting for 12 h. The tail venous blood of rats was collected by heparinized capillary blood vessels, and the blood glucose was measured by using a glucose meter (Accu-Chek; Roche Diagnostics GmbH, Germany) at 0 min before glucose loading and at 15, 30, 60, and 120 min after glucose loading. For the measurement of insulin, the rats were first allowed to fast overnight, and blood was collected on an empty stomach. After anesthetized and sacrificed, the serum samples were collected from the abdominal aorta, and insulin was determined according to the instructions of the insulin ELISA Kit (ER010r, ExCell Biotech, China).

### Metabolic Cage

Two weeks before the termination of the experiment, the rats were placed in the metabolic cage (SA104, Jiangsu SANS Biological Technology Co. Ltd., China), and the 24-h drinking water, food intake, activity, and urine collection were monitored. Besides, food consumption was evaluated throughout the study to detect any change, as previously described (Frenay et al., 2015).

### Echocardiography

One week before termination, the M-mode and two-dimensional echocardiography was performed using a high-frequency, high-resolution digital imaging system (Vevo® 2100 Imaging System, FUJIFILM VisualSonics Inc., Canada) equipped with a frequency range of 18–38 MHz for serially assessing of the cardiac structure and function. The measurements included left ventricular internal diameter at the end diastole (LVIDd), and left ventricular internal diameter at the end systole (LVIDs). The left ventricular ejection fraction (LVEF) was calculated by using the Teichholz method of estimated LV volumes. Left ventricular fractional shortening (LVFS) was to evaluate the systolic function of rats, and the calculation formula was LVFS% = (LVIDd–LVIDs)/LVIDd*100.

### Advanced Oxidation Protein Product and ATP Measurement

According to the manufacturer’s instructions, an appropriate amount of left ventricular myocardial tissue samples were taken for homogenization. Then, the supernatant was collected for AOPP and ATP measurements *via* using the AOPP assay kit (#ab242295, Abcam, United Kingdom) and ATP assay kit (#ab83355, Abcam, United Kingdom), as previously described (Yurista et al., 2019). Results were then normalized by protein concentrations of each test.

### RNA Preparation and Quantitative Real-Time

The methods used for RNA preparation and analysis were performed, as previously described (Yang et al., 2020). Briefly, total RNA of LV myocardial tissues were isolated using an RNA isolation kit (DP419, TIANGEN Biotech, China), according to the manufacturer’s protocol. Reverse transcription synthesis cDNA was amplified by SYBR Green SuperReal PreMix Plus (FP205, TIANGEN Biotech, China) by real-time fluorescent quantitative PCR amplification on an ABI 7500 PCR system (Applied Biosystems, United States). Primers were synthesized by Anhui General Biology Co., Ltd. and are listed in Table 1.

**TABLE 1:** Primers used for qRT-PCR.

Gene	Forward (5′-3′)	Reverse (5′-3′)
SIRT1	GCA​GGT​TGC​AGG​AAT​CCA​AA	TGG​CTT​CAT​GAT​GGC​AAG​TG
PGC-1a	AGA​ACC​ATG​CAA​ACC​ACA​CC	TTG​GTG​TGA​GGA​GGG​TCA​TC
β-MHC	TCT​GGA​GGC​CTT​TGG​CAA​TG	GAT​GCC​AAC​TTT​CCT​GTT​GC
α-MHC	GAC​AAC​TCC​TCC​CGC​TTT​GG	AAG​ATC​ACC​CGG​GAC​TTC​TC
β-actin	GCTCCGGCATGTGCAAAG	CCT​TCT​GAC​CCA​TTC​CCA​CC

SIRT1, sirtuin 1; PGC-1a, peroxisome proliferator-activated receptor gamma coactivator-1, alpha.

### Western Blotting

Western blotting analyses of tissues were performed, as previously described (Osataphan et al., 2019). First, 0.1 g of rat LV myocardial tissues were weighed, and total protein was extracted from the tissues. Then, the protein concentration was measured by the bicinchoninic acid (BCA) assay. The prepared same amount of protein was separated by electrophoresis with 10% sodium dodecyl sulfate-polyacrylamide gel and transferred to a PVDF membrane. After being blocked with 5% non-fat emulsion for 2 h, the PVDF membranes were incubated with primary antibodies: p-AMPK (1:1,000, Affinity Biosciences, United States), AMPK (1:1,000, Affinity Biosciences, United States), PGC-1a (1:1,000, Abcam, United Kingdom), SIRT1 (1:2000, Abcam, United Kingdom), NOX4 (1:1,000, Zen BioScience, China), β-actin (1:5,000, Zen BioScience, China), Pdk4 (1:1,000, Affinity Biosciences, United States), Ndufb4 (1:1,000, Abcam, United Kingdom), Bdh1 (1:1,000, Abcam, United Kingdom), Acox1 (1:1,000, Affinity Biosciences, United States), Ehhadh (1:1,000, Affinity Biosciences, United States) and, Acadsb (1:1,000, Abcam, United Kingdom). After washing three times with TBST, PVDF membranes were incubated with their secondary antibodies for 2 h. Signals were detected by WB chemiluminescent gel imaging (MiniChemi 610 Plus, Beijing Saizhi Venture Technology Co., Ltd., China), and band intensities were quantified by ImageJ software (National Institutes of Health, Bethesda, United States).

### Histology Analysis

After the DSS rats were anesthetized, the heart was quickly removed and weighed. The mid-papillary slice of the LV was fixed in 4% formaldehyde and paraffin-embedded. The extent of interstitial fibrosis was calculated on the Masson trichrome-stained section. Finally, heart samples were frozen and cut into 5-mm sections, which were stained with FITC-labeled wheat germ agglutinin (WGA) to determine the cardiomyocyte size. The immunohistochemical staining (IHC) of p-AMPK, PGC-1a, and SIRT1 was also performed.

### LC-MS/MS Metabolomics Analysis

Briefly, rat myocardial samples and extracts were added into an EP tube, and the supernatant was collected after grinding and centrifugation. The resulting supernatant was transferred to a fresh glass vial for analysis. LC-MS/MS analyses were performed using an UHPLC system (Vanquish, Thermo Fisher Scientific) with a UPLC BEH amide column (2.1 mm × 100 mm, 1.7 μm) coupled to a Q Exactive HFX mass spectrometer (Orbitrap MS, Thermo). The QE HFX mass spectrometer was used for its ability to acquire MS/MS spectra on the information-dependent acquisition (IDA) mode in the control of acquisition software (Xcalibur, Thermo). The raw data were converted to the mzXML format using ProteoWizard and processed with an in-house program, which was developed using R and based on XCMS, for peak detection, extraction, alignment, and integration. Then, an in-house MS2 database (BiotreeDB) was applied in metabolite annotation. The cutoff for annotation was set at 0.3. The differentially expressed metabolites should meet the importance of the first principal component variable in the OPLS-DA model at projection VIP>1 and *p* < 0.05. The metabolic pathways involved in different metabolites were analyzed on the Kyoto Encyclopedia of Genes and Genomes (KEGG) website.

### Tandem Mass Tag-Based Proteomics Analysis

Each group had three mixed samples with each containing three random myocardial samples. Rat myocardial tissues were lysed, and the supernatant was collected after centrifugation. The total protein concentration of the corresponding samples was determined by the BCA method. Following digesting, the proteins by sequence-grade modified trypsin (Promega, Madison, WI), and the resultant peptide mixture was labeled based on the TMT kit (Thermo Fisher Scientific, United States). Peptides were separated and analyzed with a nano-UPLC (EASY nLC1200) system coupled to a Q Exactive HFX Orbitrap instrument (Thermo Fisher Scientific) with a nano-electrospray ion source. Differentially expressed proteins were selected according to the three-fold change (fold change<0.83 or >1.2) and *p* < 0.05 and unique peptide ≥1. The gene ontology (GO) annotation was used to annotate the function of proteins, and KEGG was used to analyze the enriched metabolic pathways of differentially expressed proteins.

### Statistical Analysis

IBM SPSS 21.0 (IBM Corp., Armonk, United States) was used for the statistical analysis. Comparisons between groups were performed by one-way analysis of variance (ANOVA). *p* < 0.05 was considered to indicate a statistically significant difference.

## Results

### Effects of Canagliflozin on Body Weight, Blood Pressure, Food and Water Intake, Urine Volume, and Heart Weight

**FIGURE 1:**
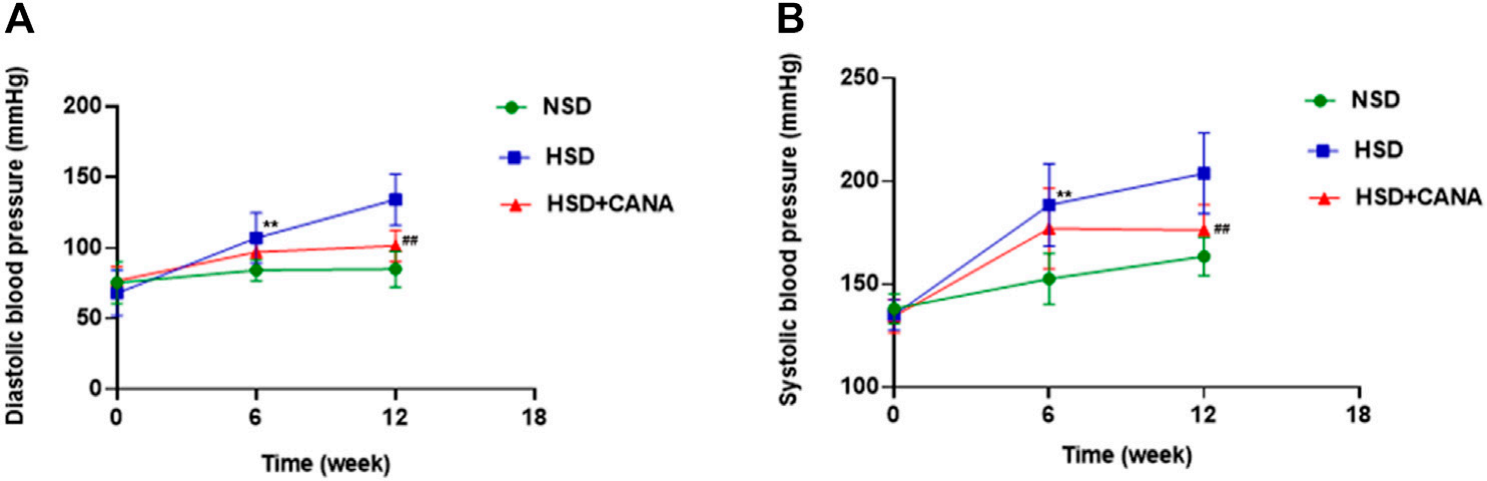
Effects of CANA on blood pressure at 0, 6, and 12 weeks. **(A)** Effect of CANA on diastolic blood pressure of DSS rats; **(B)** Effect of CANA on systolic blood pressure of DSS rats. CANA, canagliflozin; NSD, normal-salt diet; HSD, high-salt diet. ^**^ *p* < 0.01 vs. NSD. ^##^ *p* < 0.01 vs. HSD.

After 12 weeks treatment of CANA, the body weight, heart weight, heart-to-body weight ratio (HW/BW), urine volume, and water and food intake of rats in NSD, HSD, and HSD + CANA groups are shown in Table 2. As expected, 12 weeks after treatment with CANA, the body weight, heart weight, and HW/BW of rats in the HSD + CANA group were significantly lower than those in the HSD group, while the urine volume, water intake, and food intake were significantly increased. In terms of blood pressure, we analyzed the trend of changes in blood pressure within 12 weeks. As presented in Figure 1, after 6 weeks of CANA treatment, the blood pressure of the HSD + CANA group was lower than that of the HSD group, but there was no statistical difference. However, after 12 weeks of treatment, systolic and diastolic blood pressure in the HSD + CANA group was significantly lower than that in the HSD group.

**TABLE 2:** Effects of CANA on physiological parameters after treatment for 12 weeks.

	NSD	HSD	HSD + CANA
HW (g)	1.21 ± 0.05	1.57 ± 0.18^**^	1.15 ± 0.12^##^
BW (g)	373.78 ± 12.46	338.83 ± 21.54^**^	305.97 ± 21.02^##^
HW/BW (mg/g)	3.25 ± 0.11	4.64 ± 0.47^**^	3.75 ± 0.35^##^
Food intake (g/24 h)	15.72 ± 2.37	18.07 ± 1.64	27.76 ± 6.58^#^
Water intake (g/24 h)	19.5 ± 7.87	82.17 ± 20.12^**^	135.17 ± 20.5^##^
Urine volume (mL/24 h)	9.58 ± 4.39	57.17 ± 20.74^**^	105.83 ± 7.81^##^

CANA, canagliflozin; NSD, normal-salt diet; HSD, high-salt diet; HW, heart weight; BW, body weight; HW/BW, heart-to-body weight ratio; ^**^ *p* < 0.01 vs. NSD. ^#^ *p* < 0.05, ^##^ *p* < 0.01 vs. HSD.

### Effects of Canagliflozin on Serum and Urine Biochemical Parameters

As shown in Table 3, after 12 weeks of high-salt diet, the urine glucose, urine sodium, and urine albumin of the rats were significantly increased, with statistical differences. After CANA intervention, the urine glucose increased and urinary albumin decreased significantly in the HSD + CANA group, as compared to the HSD group. Meanwhile, we observed that serum creatinine and urinary creatinine of the HSD group were significantly increased compared with the NSD group. After 12 weeks of CANA treatment, both serum creatinine and urinary creatinine decreased; only urinary creatinine had a statistical difference (Table 3). There was no statistical difference in serum albumin, serum sodium, total cholesterol, LDLC, and triglycerides among the three groups. Besides, CANA significantly increased the concentration of 3-hydroxybutyric acid in the blood circulation of DSS rats.

**TABLE 3:** Effects of CANA on blood and urine measurements after treatment for 12 weeks.

	NSD	HSD	HSD + CANA
Urine glucose (mmol/L)	0.05 ± 0.02	0.13 ± 0.05^*^	45.55 ± 13.54^##^
Urine sodium (mmol/L)	21.01 ± 5.75	154.42 ± 76.16^*^	119.36 ± 29.06
Urine albumin (g/L)	0.39 ± 0.22	1.82 ± 0.45^**^	0.97 ± 0.36^##^
Urinary creatinine (μmol/L)	6,558.62 ± 4,348.19	10,068.16 ± 3,573.02^**^	2,364.76 ± 1,055.72^#^
Serum albumin (g/L)	30.11 ± 1.56	28.19 ± 2.52	27.96 ± 1.93
Serum sodium (mmol/L)	174.41 ± 14.35	160.47 ± 15.54	175.52 ± 13.24
Serum creatinine (μmol/L)	27.48 ± 4.07	40.06 ± 7.57^**^	33.37 ± 4.01
Total cholesterol (mmol/L)	2.54 ± 0.39	3.47 ± 0.97	3.34 ± 1.08
LDLC (mmol/L)	1.43 ± 0.15	1.99 ± 0.42	1.7 ± 0.52
Triglyceride (mmol/L)	0.48 ± 0.16	0.6 ± 0.24	0.55 ± 0.28
3-hydroxybutyric acid (mmol/L)	1.05 ± 0.08	1.06 ± 0.04	1.36 ± 0.12^##^

CANA, canagliflozin; NSD, normal-salt diet; HSD, high-salt diet; LDLC, low-density lipoprotein cholesterol; ^*^ *p* < 0.05, ^**^ *p* < 0.01 vs. NSD. ^#^ *p* < 0.05, ^##^ *p* < 0.01 vs. HSD.

### Effect of Canagliflozin on Cardiac Function

**FIGURE 2:**
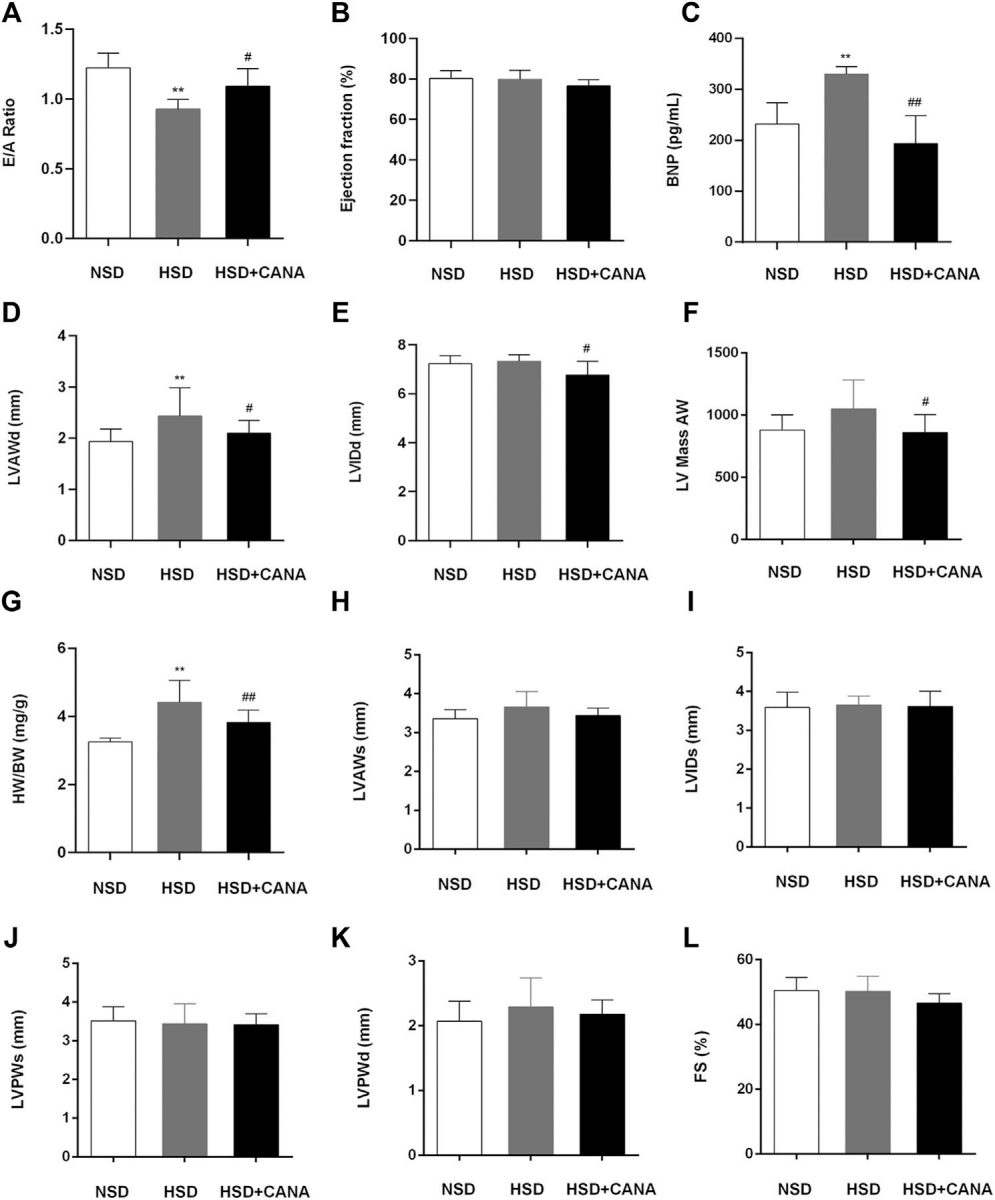
Effect of CANA on cardiac function. **(A)** Evaluation of the ratio of peak E to peak A (E/A) by echocardiography; **(B)** Left ventricular ejection fraction (EF); **(C)** Concentration of BNP in the serum; **(D)** Left ventricular anterior wall end diastole (LVAWd); **(E)** Left ventricular internal diameter end diastole (LVIDd); **(F)** Left ventricular mass AW; **(G)** Heart-to-body weight ratio (HW/BW); **(H)** Left ventricular anterior wall end systole (LVAWs); **(I)** Left ventricular internal diameter end systole (LVIDs); **(J)** Left ventricular posterior wall end systole (LVPWs); **(K)** Left ventricular posterior wall end diastole (LVPWd); **(L)** Fractional shortening (FS); CANA, canagliflozin; NSD, normal-salt diet; HSD, high-salt diet. ^**^ *p* < 0.01 vs. NSD. ^#^ *p* < 0.05, ^##^ *p* < 0.01 vs. HSD.

To investigate the effect of CANA on cardiac function, echocardiography was performed, and relevant indicators are presented in Figure 2. After 12 weeks of high-salt diet, the average ratio of peak E to peak A (E/A) was 0.93 ± 0.07 and LVEF was 79.91 ± 4.45 in the HSD group, suggesting the presence of LV diastolic dysfunction (Figures 2A,B). The detected serum BNP showed that the BNP in the HSD group was significantly increased, and it was considered that there may be cardiac insufficiency (Figure 2C). Interestingly, after CANA treatment, E/A in the HSD + CANA group increased significantly, indicating that CANA improved the diastolic function of rats. Meanwhile, we also observed that CANA intervention evidently reduced BNP levels, as compared to the HSD group. In addition, hypertension was observed to lead to LV hypertrophy. However, CANA treatment evidently reduced the LVAWd, LVIDd, and LV Mass AW in the HSD + CANA group, as compared to the HSD group (Figures 2D–F). Consistently, CANA significantly reduced HW/BW in the HSD + CANA group (Figure 2G). There was no statistical difference in LVEF, LVAWs, LVIDs, LVPWs, LVPWd, and FS measured by ultrasound among the three groups (Figures 2B,H–L).

### Effect of Canagliflozin on Cardiac Histology and Molecular Markers for Remodeling and Fibrosis

**FIGURE 3:**
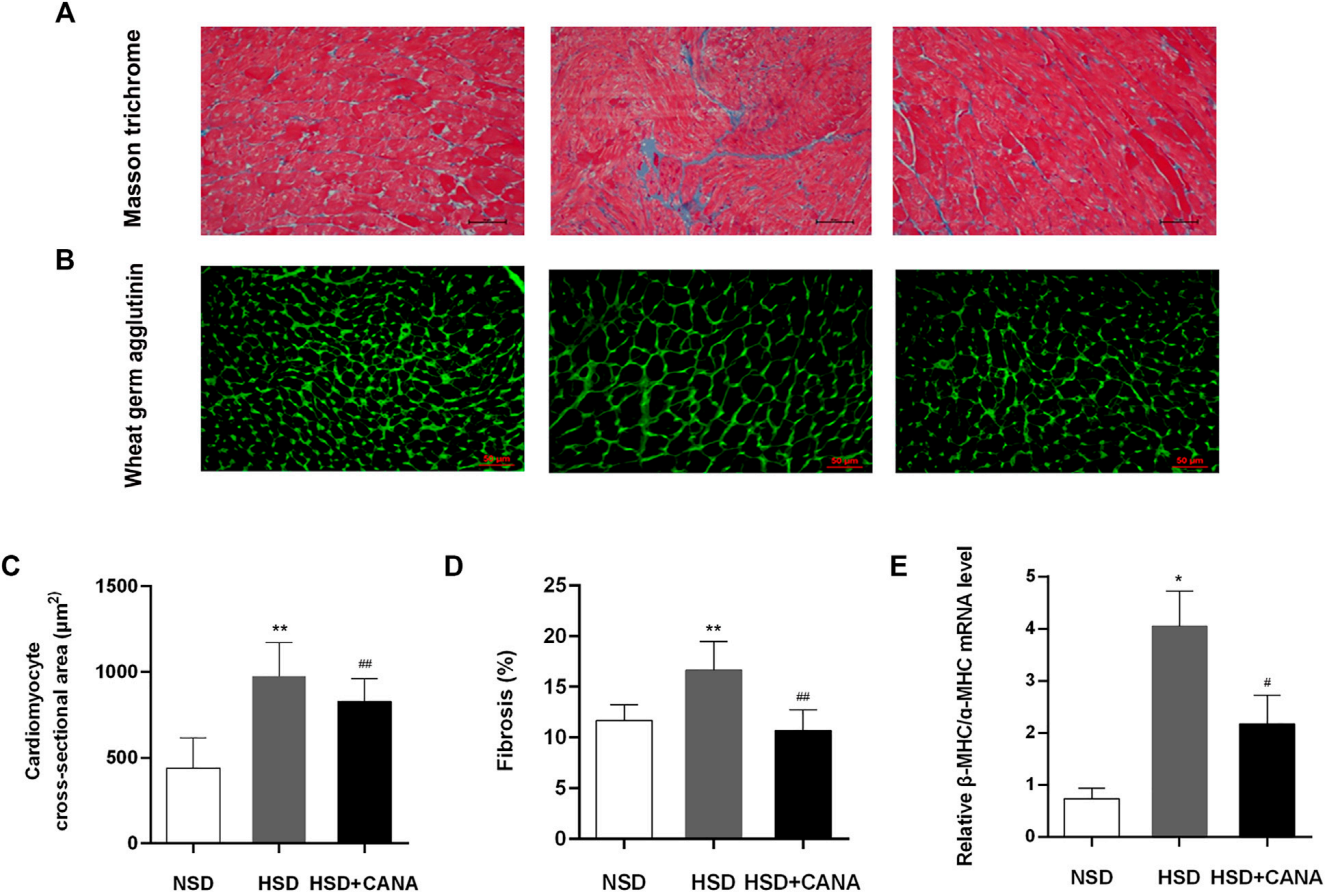
Effect of CANA on cardiac histology and molecular markers for remodeling and fibrosis. **(A)** Representative left ventricular sections stained by Masson trichrome staining; **(B)** Representative left ventricular sections stained by WGA staining; **(C)** Quantification of the cardiomyocyte cross-sectional area from the wheat germ agglutinin (WGA)-stained section; **(D)** Quantification of fibrosis in the left ventricle from the Masson trichrome-stained section; **(E)** Measurement of mRNA levels to assess molecular markers for remodeling and fibrosis. NSD, normal-salt diet; HSD, high-salt diet. ^*^ *p* < 0.05, ^**^ *p* < 0.01 vs. NSD. ^#^ *p* < 0.05, ^##^ *p* < 0.01 vs. HSD.

In Figures 3A,B, we observed that a high-salt diet resulted in LV hypertrophy, which was manifested as the increased cross-sectional area of myocardial cells. However, after 12 weeks of CANA treatment, the cross-sectional area of myocardial cells in the HSD + CANA group was significantly reduced, which was consistent with the reduction in the LV mass (Figures 3B,C). Meanwhile, we also observed a significant increase in LV interstitial fibrosis in the HSD group, and the degree of fibrosis was significantly reduced after CANA treatment (Figures 3A,D). The high ratio of myosin heavy chain subtypes β-MHC to ɑ-MHC is usually considered a sign of cardiac hypertrophy (Cox and Marsh, 2014). In Figure 3E, a high-salt diet increased the ratio of β-MHC/ɑ-MHC and decreased after CANA intervention, which was close to the NSD group.

### Effects of Canagliflozin on Blood Glucose, Oxidative Stress, and Cardiac ATP Production

**FIGURE 4:**
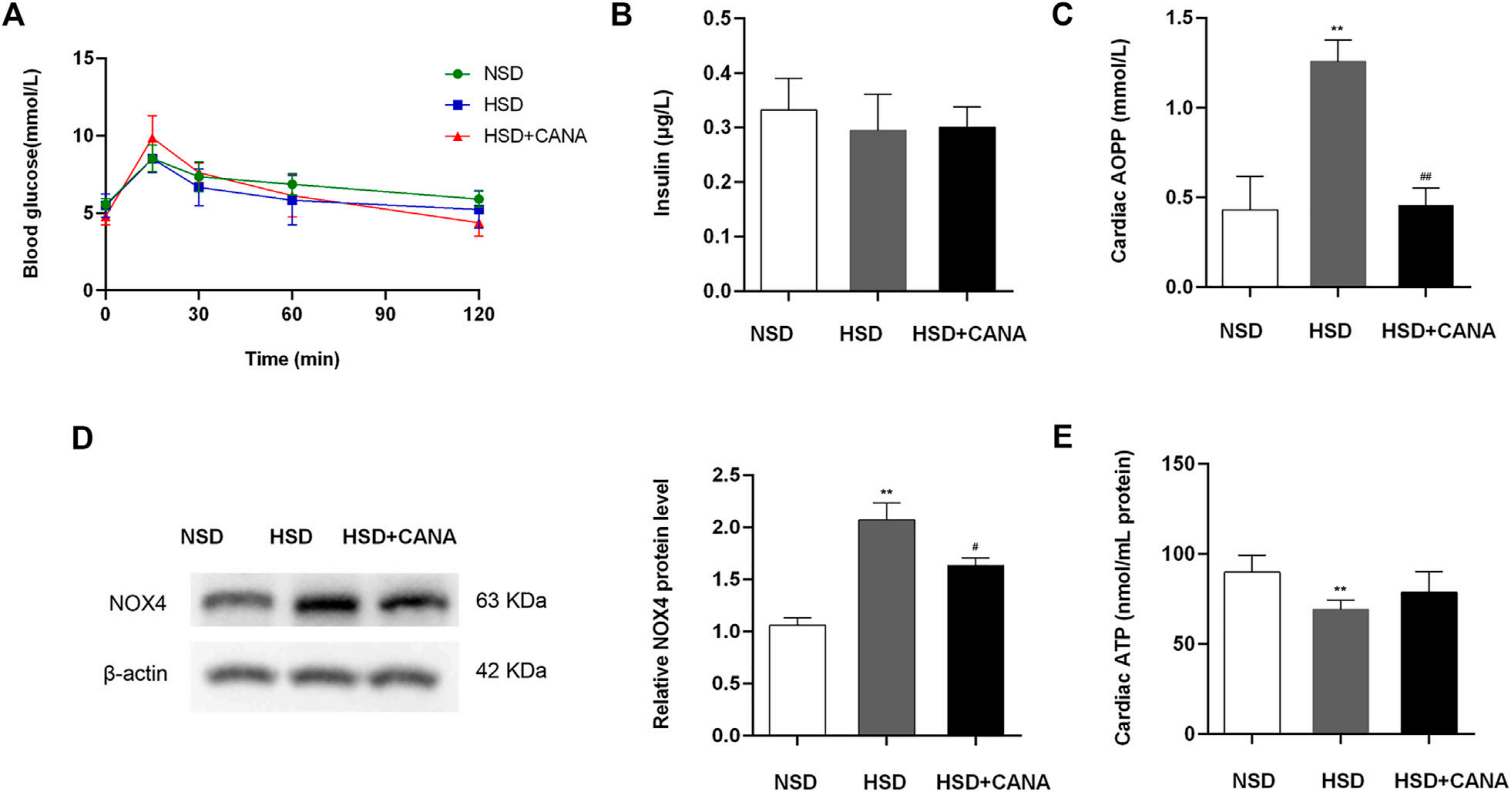
Effects of CANA on blood glucose, oxidative stress, and cardiac ATP production. **(A)** Oral glucose tolerance (2 mg/kg) after overnight fasting for 12 h; **(B)** Serum insulin after overnight fasting for 12 h; **(C)** Cardiac advanced oxidation protein products to assess oxidative stress; **(D)** Measurement of NOX4 protein levels; **(E)** Cardiac ATP production was determined in indicated groups. NSD, normal-salt diet; HSD, high-salt diet. ^**^ *p* < 0.01 vs. NSD. ^#^ *p* < 0.05, ^##^ *p* < 0.01 vs. HSD.

CANA is a hypoglycemic drug; therefore, we evaluated its effect on blood sugar in non-diabetic rats. OGTT results showed that the area under the blood glucose curve of the HSD + CANA group was not statistically different from the other two groups, and insulin was also not statistically different among the three groups (Figures 4A,B). As oxidative stress is closely related to myocardial fibrosis, the influence of CANA on myocardial oxidative stress was determined. In Figures 4C,D, we found that a high-salt diet induced the expression of AOPP and NOX4 protein, but they were significantly reduced after CANA treatment. Meanwhile, we also tested the ATP level in each group. Compared with the NSD group, the ATP level of the HSD group was evidently reduced. After 12 weeks of CANA treatment, the ATP levels in the HSD + CANA group showed an upward trend (Figure 4E).

### Effect of Canagliflozin on Metabolic Pathways

**FIGURE 5:**
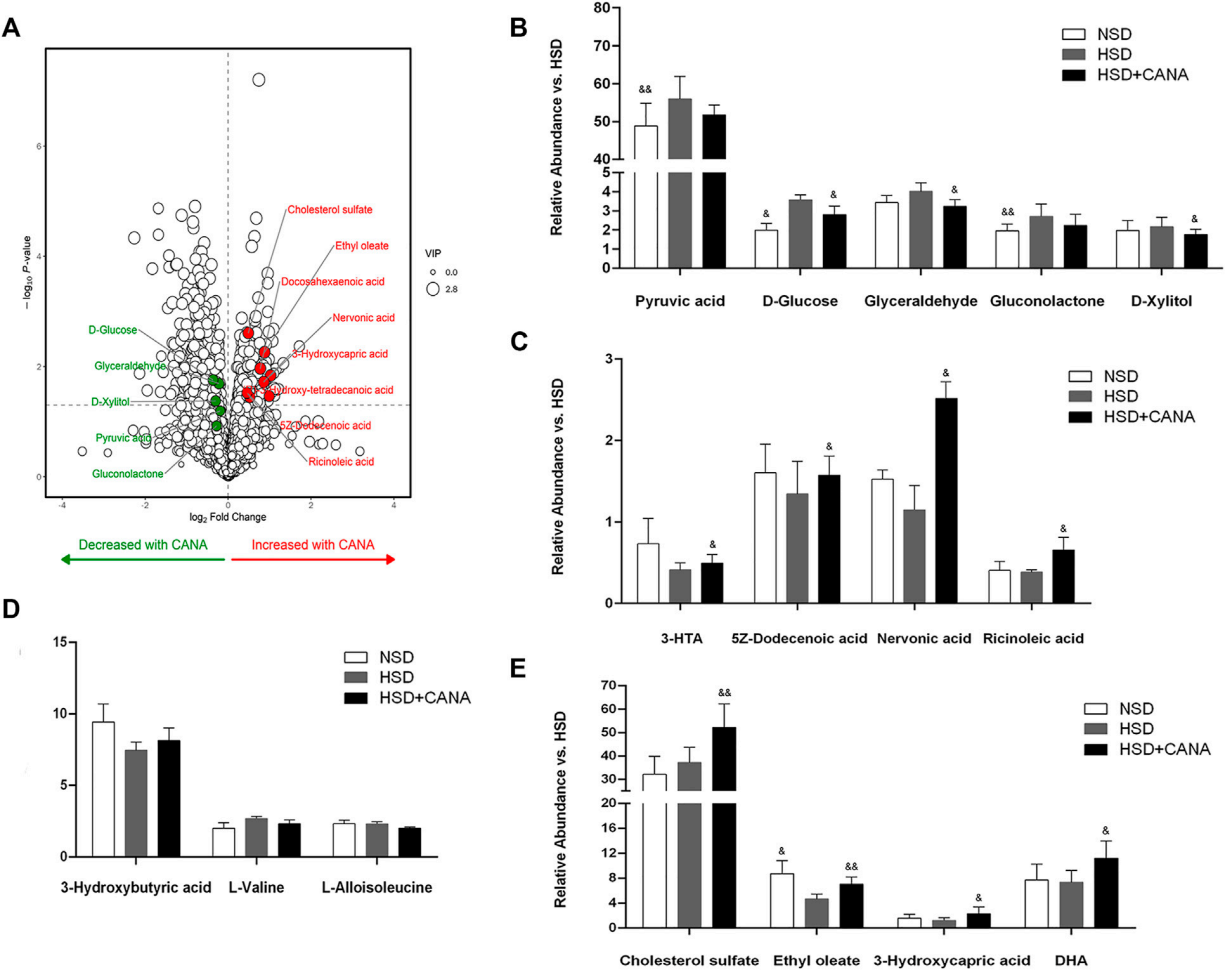
Effect of CANA on metabolic pathways. **(A)** Volcano plot of cardiac metabolites altered by CANA, in comparison to HSD; **(B–E)** Representative catabolic metabolites. NSD, normal-salt diet; HSD, high-salt diet; 3-HTA, 3-hydroxy-Tetradecanoic acid; DHA, docosahexaenoic acid; ^&^ *p* < 0.05, ^&&^ *p* < 0.01 vs. HSD.

Next, we evaluated the effect of CANA on myocardial metabolic pathways. Myocardial tissue metabolomics analysis showed that compared with the HSD group, the concentrations of various myocardial metabolites in the HSD + CANA group were changed. When the screening conditions were set to VIP > 1 and *p* < 0.05, 34 metabolites of the 166 identified metabolites were changed significantly (Figure 5A). After CANA treatment, glucose metabolites, including pyruvic acid, D-glucose, glyceraldehyde, gluconolactone, and D-xylitol, were reduced as compared to the HSD group, of which D-glucose, glyceraldehyde, and D-xylitol were statistically significant (Figure 5B). In contrast, oxidative metabolic degradation products of fatty acids such as 3-hydroxy-Tetradecanoic acid (3-HTA),5Z-dodecenoic acid, nervonic acid, ricinoleic acid, ethyl oleate, cholesterol sulfate, 3-hydroxycapric acid, and docosahexaenoic acid (DHA) were increased significantly (Figures 5C,E). Among them, 5Z-dodecenoic acid, ricinoleic acid, and ethyl oleate are monounsaturated fatty acids. More interestingly, we observed that the polyunsaturated fatty acid DHA increased after CANA treatment, but it is not a fatty acid oxidation metabolite of the rat myocardium because the rat myocardium lacks elongase-2 and cannot synthesize DHA and needs to be obtained from the plasma (Igarashi et al., 2008). Consistent with the increase in serum ketone bodies, after CANA treatment, 3-hydroxybutyric acid was increased in the myocardium with no statistically significant difference compared with the HSD group. Besides, we also observed that L-valine and L-alloisoleucine in the HSD + CANA group were less than those in the HSD group, but there was still no statistical difference (Figure 5D).

### Effect of Canagliflozin on the Cardiac Protein Expression

**FIGURE 6:**
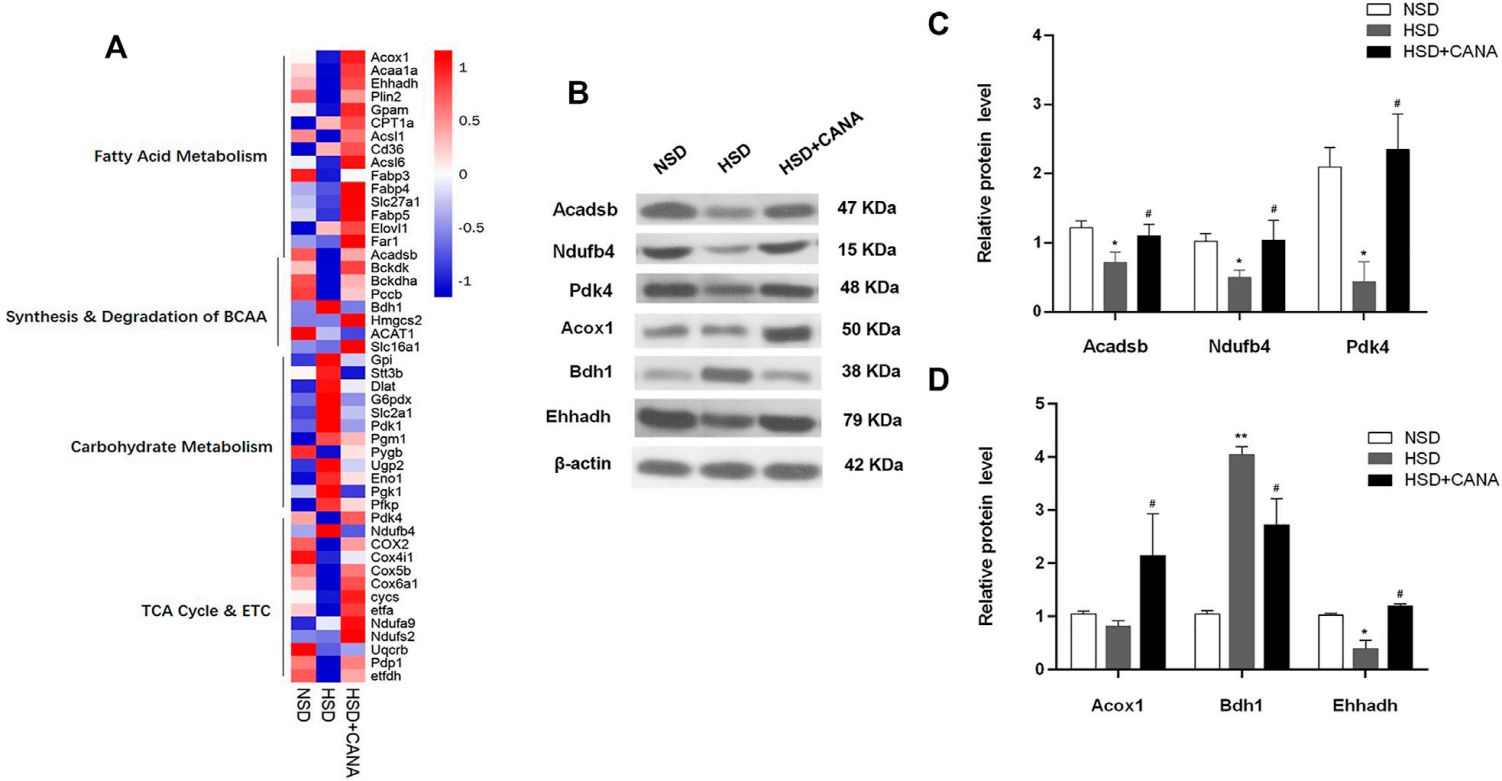
Effect of CANA on the cardiac protein expression. **(A)** Heat map of individual genes within selected pathways, colored by the log2fold change; **(B)** Selected genes (Acadsb, Ndufb4, Pdk4, Acox1, Bdh1, and Ehhadh) were validated by Western blotting; **(C,D)** Quantitative evaluation of the protein expression of selected genes (Acadsb, Ndufb4, Pdk4, Acox1, Bdh1, and Ehhadh). ^*^ *p* < 0.05, ^**^ *p* < 0.01 vs. NSD. ^#^ *p* < 0.05 vs. HSD.

To further study the changes of CANA on myocardial metabolic pathways, we performed non-targeted proteomics analysis on the myocardium of the three groups. After CANA treatment, compared with the HSD group, a total of 85 proteins were significantly changed. In carbohydrate metabolism, Gpi, Dlat, G6pdx, Slc2a1, Pgm1, Pygb, Ugp2, and other proteins were downregulated, but there was no significant statistical difference. Only the Stt3b protein involved in glycosylation was significantly decreased (Figure 6A). In the TCA cycle and ETC, 13 related proteins were upregulated, among which Pdk4, Ndufb4, COX2, Cox4i1, Cox5b, and Cox6a1 were significantly increased, especially Pdk4 (Figures 6A–C). In addition, we observed a significant increase in the expression of mitochondrial β-oxidation and ketogenic proteins, including Acox1, Acaa1a, Ehhadh, Plin2, Acadsb, Gpam, and Hmgcs2. Of note is the fact that the expression of Bdh1 related to ketone metabolism was increased in HSD but significantly decreased after CANA treatment. Coupled with the increase of 3-hydroxybutyric acid in the serum and myocardium, it suggested that CANA may not improve the utilization of cardiac ketones (Figures 6A–D). Consistent with the metabolomics changes, the key enzymes Bckdk and Bckdha in the branched-chain amino acid degradation pathway showed an increased trend after CANA treatment, but there was no significant statistical difference, while the Pccb protein related to isoleucine metabolism in branched-chain amino acids was significantly upregulated (Figure 6A).

### CANA Improves Cardiac Function by Activation of Fatty Acid Oxidation and Ketogenesis and Reducing Oxidative Stress Through the AMPK/SIRT1/PGC-1a Pathway

**FIGURE 7:**
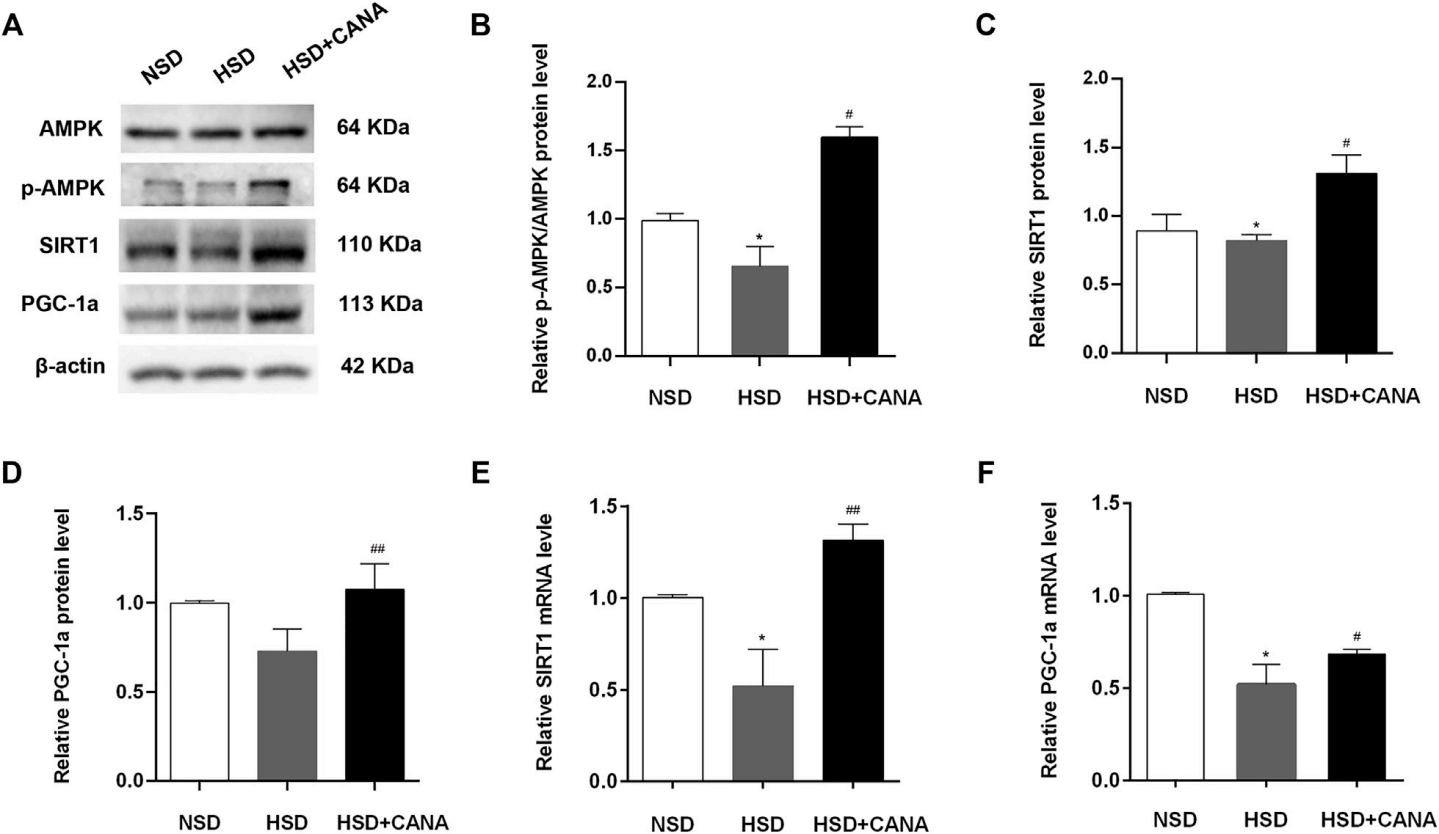
CANA improves the cardiac function by activation of fatty acid oxidation and ketogenesis and reducing oxidative stress through the AMPK/SIRT1/PGC-1a pathway. **(A)** The protein expression of AMPK, p-AMPK, SIRT1, and PGC-1a was detected *via* Western blotting; **(B)** Quantitative evaluation of the protein expression of AMPK and p-AMPK; **(C)** Quantitative evaluation of the protein expression of SIRT1; **(D)** Quantitative evaluation of the protein expression of PGC-1a; **(E)** The mRNA expression of SIRT1 was detected in indicated groups; **(F)** The mRNA expression of PGC-1a was detected in indicated groups. ^*^ *p* < 0.05 vs. NSD. ^#^ *p* < 0.05, ^##^ *p* < 0.01 vs. HSD.

**FIGURE 8:**
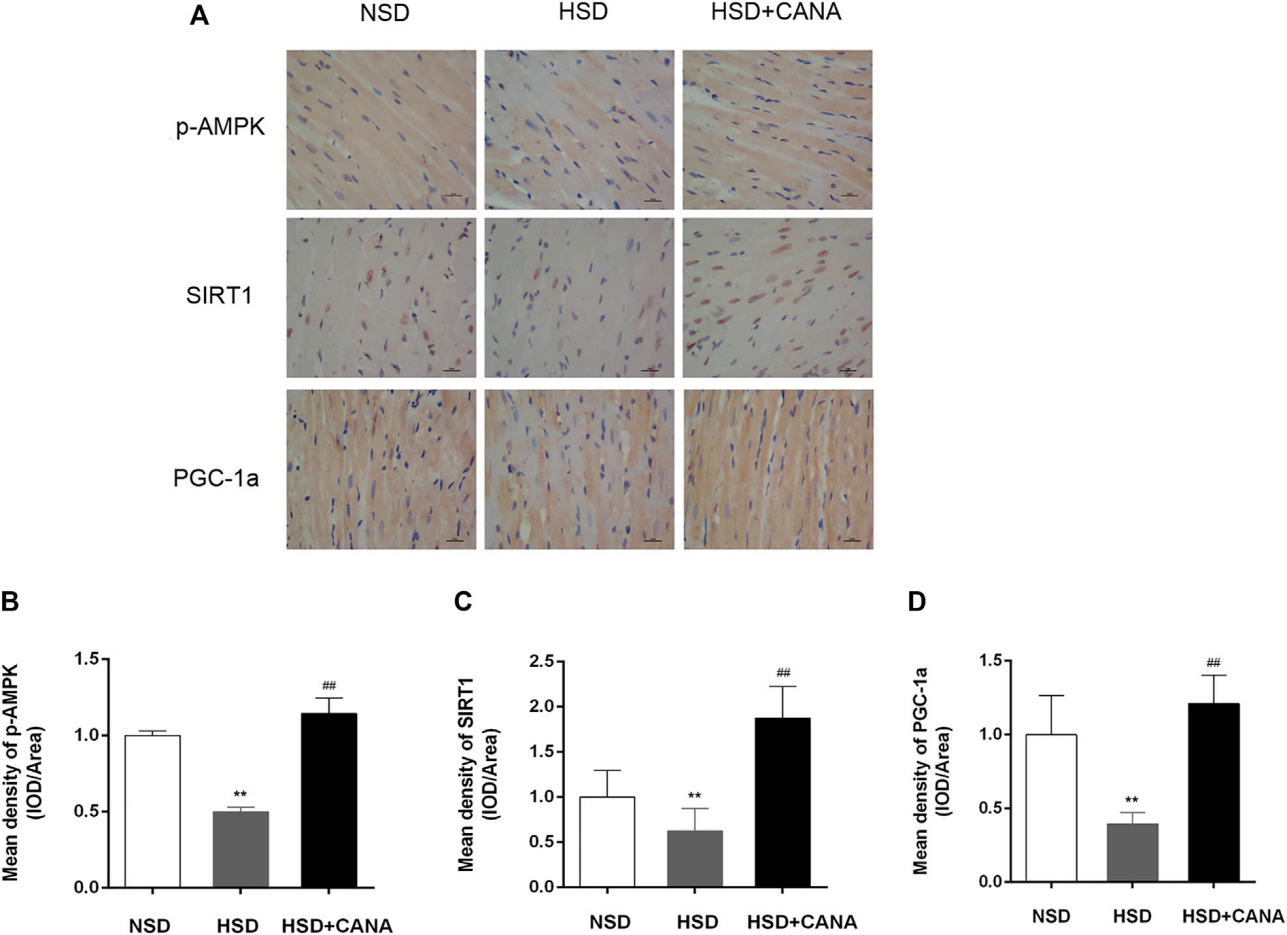
CANA improved cardiac function by activation of fatty acid oxidation and ketogenesis and reducing oxidative stress through the AMPK/SIRT1/PGC-1a pathway. **(A)** The protein expression of AMPK, p-AMPK, SIRT1, and PGC-1a was detected *via* immunohistochemical staining; **(B)** Mean density of p-AMPK (IOD/Area); **(C)** Mean density of SIRT1 (IOD/Area); **(D)** Mean density of PGC-1a (IOD/Area); IOD, integrated optical density. ^**^ *p* < 0.01 vs. NSD. ^##^ *p* < 0.01 vs. HSD.

The increase of ketone bodies and DHA in the blood circulation and myocardium can promote the SIRT1 expression (Jung et al., 2013; Saboori et al., 2016). To investigate whether SIRT1 is a key protein that coordinates CANA-mediated changes in myocardial metabolism, we further examined the expression of SIRT1 and interacting protein (p-AMPK) and downstream (PGC-1α) regulatory mediators. As depicted in Western blot data, they revealed that CANA intervention obviously induced the expression of p-AMPK, SIRT1, and PGC-1α proteins (Figure 7A). Correspondingly, the quantitative data showed a significant increase of p-AMPK, SIRT1, and PGC-1α protein levels after CANA intervention, compared to the HSD group (Figures 7C,D). In addition, at the transcriptional level, we also observed that mRNA levels of SIRT1 and PGC-1α were remarkably increased after CANA treatment, compared with the HSD group (Figures 7E,F). Furthermore, we performed IHC to strengthen the verification. As shown in Figure 8, compared with the HSD group, the p-AMPK, SIRT1, and PGC-1α protein levels of the HSD + CANA group were significantly increased, which was consistent with the results of Western blotting.

## Discussion

DSS rats, when fed an 8% NaCl diet, will develop renal failure, severe hypertension, and left ventricular hypertrophy. After 5–6 weeks of continuous feeding, the blood pressure will increase significantly, and cardiac diastolic dysfunction will occur in rats at 12 weeks (Kim-Mitsuyama et al., 2004). At 16–20 weeks of the high-salt diet, cardiac enlargement and a decrease in LVEF gradually occur, leading to progression to the HFrEF (Klotz et al., 2006). However, differences in experimental conditions, such as the specific genetic background of the animals and the environmental conditions of the rearing environment, including diet, may have a significant impact on the cardiovascular phenotype of DSS rats, and the time to disease progression is also inconsistent. In our study, DSS rats were fed with 8% high-salt diet for 5–6 weeks, and blood pressure was significantly increased. After continuing high-salt feeding for 12 weeks, the HFpEF gradually developed, which was manifested as the increased LV mass, increased BNP, and E/A <1 but EF>60%. This was similar to what was observed in other trials that DSS was in the HFpEF after 12 weeks of 8% high-salt feeding. After 12 weeks of CANA treatment, the urine volume and the systolic and diastolic blood pressure were significantly reduced. Interestingly, CANA intervention did not cause low blood sodium and even evidently reduced urine creatinine and urine protein and improved kidney function. Empagliflozin has been shown to improve the cardiac function, cardiac hypertrophy, and fibrosis in non-diabetic rats with left ventricular dysfunction after myocardial infarction and promote the normalization of myocardial glucose and fatty acid oxidation metabolism and cardiac ATP production (Yurista et al., 2019). Similarly, we also confirmed that CANA treatment can prevent LV remodeling caused by the high-salt diet in DSS rats, as well as reduce myocardial hypertrophy and fibrosis, and improve LV diastolic dysfunction. Although ATP increased after CANA intervention, there was no statistical difference, which might be related to the progression of HF. In addition, there was no reduction of EF and complete decompensation of the heart in our study, which may be due to the short observation period of experimental drugs. Notably, SGLT2 inhibitors have also been observed to improve diastolic function, but not systolic function, in other models of HFpEF (Pabel et al., 2018; Park et al., 2020), similar to our results. Yurista et al. reported that in non-diabetic rats, empagliflozin improved the cardiac function with LV dysfunction after myocardial infarction without affecting renal function and maintaining normal plasma glucose and electrolyte levels (Yurista et al., 2019). As expected, compared with the HSD group, the blood glucose of the HSD + CANA group did not decrease significantly, and there was no statistical difference in the blood insulin concentration and the area under the OGTT curve, suggesting that CANA did not cause a further decrease in blood glucose in rats with normal blood sugar. However, CANA intervention triggered a metabolic conversion from using glucose carbon as fuel to a fasting state, which increased the utilization of fatty acids and ketogenesis, and caused polyuria, polydipsia, and weight loss.

In contrast to weight loss caused by diet, the energy expenditure during weight loss caused by the SGLT2i did not decrease (Ferrannini et al., 2015), which may be due to increased lipid oxidation (Osataphan et al., 2019). With the reduction of glucose and its utilization, alternative fuels must be used to provide the TCA cycle during fasting, such as amino acids, fatty acids, or ketone bodies (Akram, 2014; Rui, 2014; Santiago-Hernandez et al., 2021). Here, we found that BCAA in the myocardium was only slightly reduced after CANA treatment, and the expression of regulatory proteins did not change significantly, suggesting that there was no significant increase in amino acid catabolism. In addition, our evidence suggested that fatty acid metabolism was a major pathway for myocardial energy metabolism transfer after CANA treatment, including fatty acid oxidation and upregulation of TCA circulating active proteins. More interestingly, CANA was observed to increase unsaturated lipids, including the significantly upregulated polyunsaturated fatty acid DHA. Although DHA is not an oxidative metabolite of cardiac fatty acids, studies have shown that DHA reduced the mitochondrial membrane viscosity, accelerated the Ca2+ uptake, and reduced the mitochondrial permeability transition and the development of LV dysfunction (Dabkowski et al., 2013). Collectively, the data revealed that the SGLT2i can upregulate the myocardial fatty acid oxidative metabolism and TCA cycle-related proteins to regulate the myocardial metabolism. Besides, CANA can increase the levels of unsaturated fatty acids and has a protective effect on the cardiovascular system.

Regardless of whether there is diabetes, SGLT2i will increase the content of 3-hydroxybutyric acid in serum and urine (Kim and Lee, 2019). Similarly, we observed that CANA significantly increased 3-hydroxybutyric acid in the blood circulation and slightly increased 3-hydroxybutyric acid in the myocardium. Ferrannini et al. proposed that the increased circulating level of 3-hydroxybutyric acid provided significant cardioprotection for high-risk diabetic patients (Jordan et al., 2016) because it was oxidized by the heart prior to fatty acids and glucose, and it can not only improve the cardiac function of patients with HF but also improve the mechanical efficiency. Besides, it is worth considering that the SGTL2i may increase the synthesis of ketones by oxidizing fatty acids and increasing the ketogenic effect. However, it cannot be ruled out that the SGLT2i may also reduce the ketone clearance rate *in vivo*. After 12 weeks of CANA treatment in DSS rats, we also observed a significant increase in the myocardial ketogenic protein Hmgcs2 but a significant decrease in the expression of Bdh1 protein related to ketone body oxidation in the myocardium. Therefore, it is speculated that the SGLT2i may actually increase serum ketone body levels through the ketogenic effect and reduce myocardial ketone body oxidation. Myocardial Hmgcs2 is the rate-limiting enzyme of ketone body production, which promotes the generation of 3-hydroxybutyric acid in the myocardium. However, study has shown that the Hmgcs2 overexpression is related to myocardial lipotoxicity caused by a high-fat diet in mice, and the inhibition of the upregulation of Hmgcs2 can reverse cardiomyopathy induced by lipotoxicity (Sikder et al., 2018; Li et al., 2021). Thus, the cardioprotective effect of SGLT2i may not be mediated by upregulation of Hmgcs2 protein. For the high expression of Bdh1 and the increased oxidation of ketone bodies in the HSD group, it may be an adaptive change in heart failure, which was also observed in other studies. For example, Bdh1-knockout mice showed more severe ventricular remodeling and dysfunction after TAC/myocardial infarction (Uchihashi et al., 2017), whereas in the stress overload model, the Bdh1 overexpression attenuated cardiac remodeling and DNA damage, suggesting that increased ketone utilization was adaptive (Shimazu et al., 2013). Taken together, we speculate that CANA may improve cardiac function through other mechanisms, such as higher 3-hydroxybutyric acid levels and increased fatty acid oxidation in the myocardium, rather than increased oxidation of ketone bodies, possibly simply to reduce the excessive Bdh1 expression and reduce ketone oxidation of the body returns to normal levels.

The cardioprotective effect of the SGLT2i may be related to its ability to induce a fasting-like paradigm, which triggers the activation of the nutrient deprivation pathway to promote cardiomyocyte homeostasis. The most unique metabolic feature of fasting is the enhanced ketogenic ability, which is not available in other hypoglycemic drugs, and significantly increased ketone bodies can increase the expression of SIRT1 (Umino et al., 2018). SIRT1 and AMPK tend to be mutually activated, and they share many common downstream targets, including PGC-1a (Cantó et al., 2009). Yang et al. demonstrated that CANA can activate the AMPK/SIRT1/PGC-1a signaling pathway (Yang et al., 2020), which was also consistent with our observation. PGC-1a is an important regulator of mitochondrial biosynthesis, and its decreased production is believed to be the cause of mitochondrial dysfunction in HF (Tanajak et al., 2018). Yurista et al. confirmed that SGLT2i can restore mtDNA damage and increase the expression of PGC-1a in non-diabetic myocardial infarction rats with the HFrEF (Yurista et al., 2019). Here, in the myocardium of the hypertension-induced HFpEF, we also observed the increased expression of PGC-1a after CANA treatment, which was consistent with the increased expression of the mitochondrial membrane respiratory chain component protein subunit Ndufb4, COX2, Cox4i1, Cox5b, and Cox6a1. These results suggested that the effects of CANA on mitochondrial biogenesis can be translated into non-diabetic HFpEF, regardless of whether the ejection fraction was reduced, most likely through activation of the AMPK/SIRT1/PGC-1a signaling pathway.

## Conclusion

CANA has a good effect on the cardiac function and remodeling of rats with LV diastolic function caused by hypertension, which was related to the increase of fatty acid oxidation and ketogenesis, and the reduction of oxidative stress, possibly mediated by activating the AMPK/SIRT1/PGC-1a signaling pathway. In addition, early administration of CANA may be beneficial in heart failure patients without diabetes, even if LVEF was in the normal range.

## Data Availability

The original contributions presented in the study are included in the article/Supplementary Material, further inquiries can be directed to the corresponding author.
