# Supplementary figures and images for: An Effective Sodium-Dependent Glucose Transporter 2 Inhibition, Canagliflozin, Prevents Development of Hypertensive Heart Failure in Dahl Salt-Sensitive Rats
Source: Front Pharmacol. 2022 Mar 9;13:856386. doi: 10.3389/fphar.2022.856386 (PMC8964360; doi:10.3389/fphar.2022.856386)

**
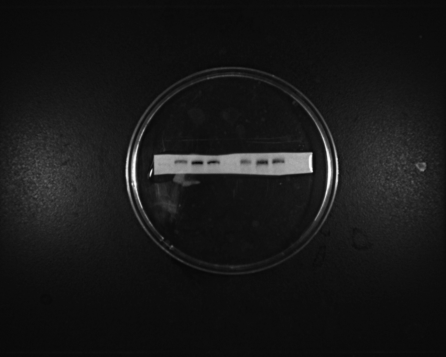
Figure 4D**

**NOX4**

**β
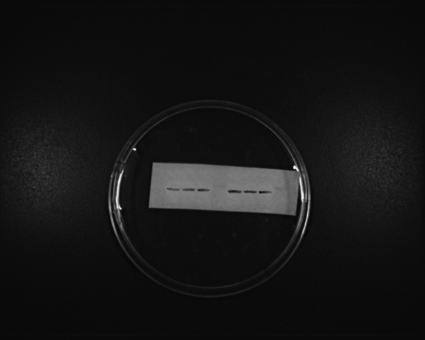
-actin**

**Figure 6**


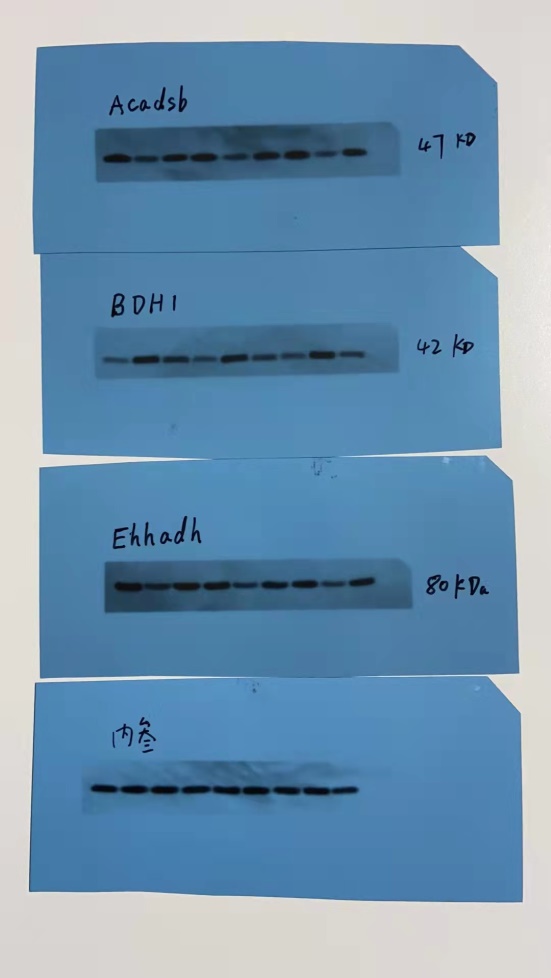


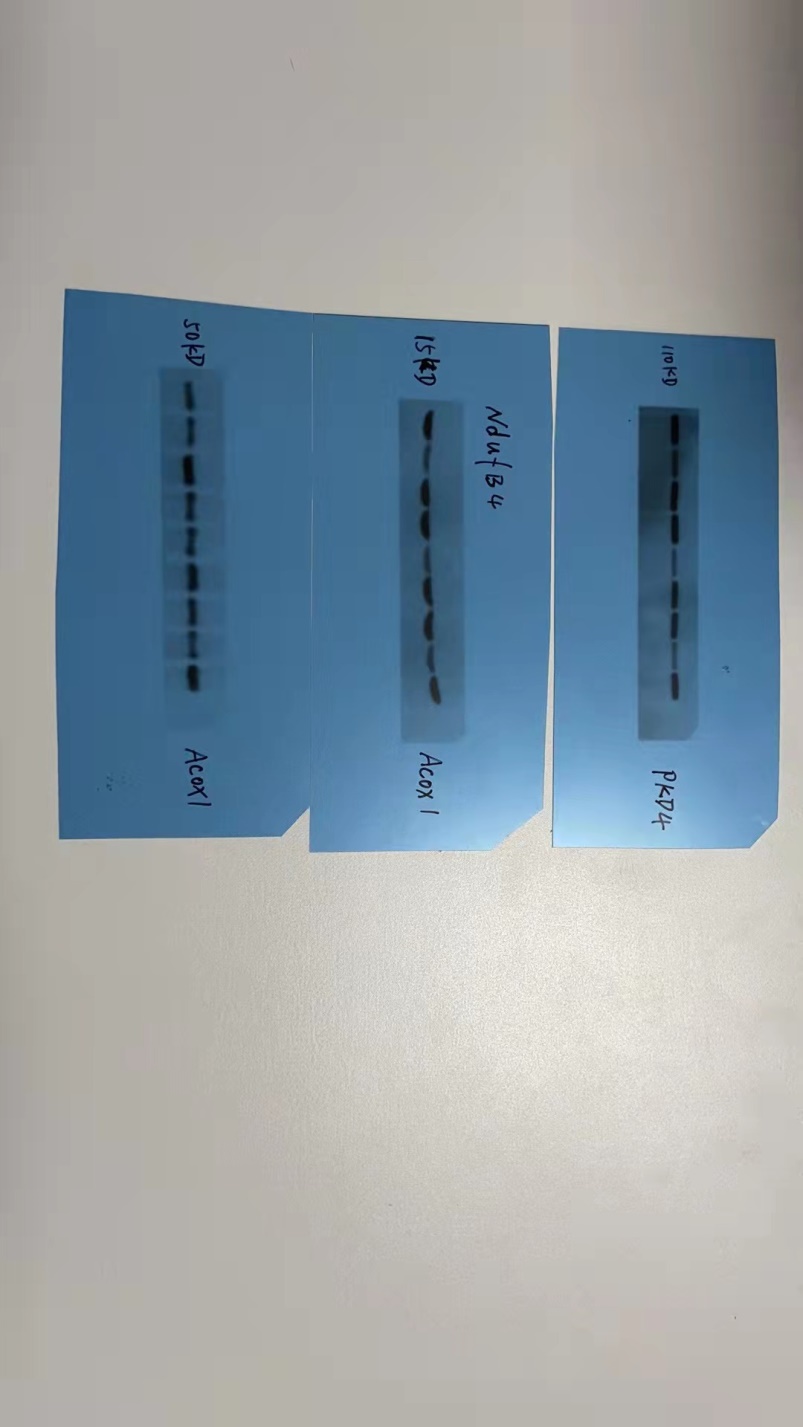


**Figure 7**


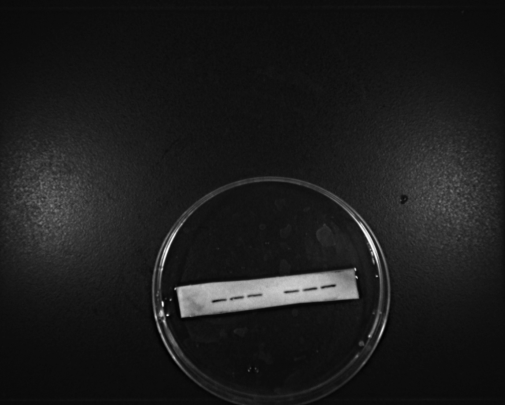
**AMPK**


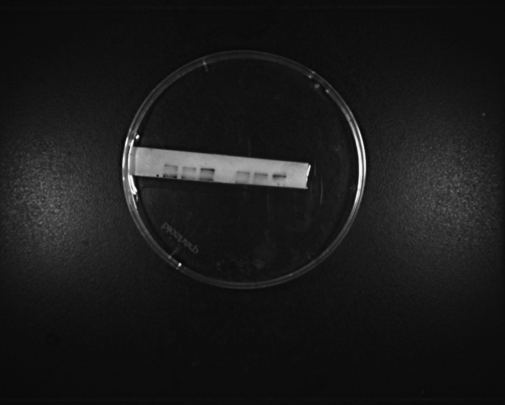
**p-AMPK**


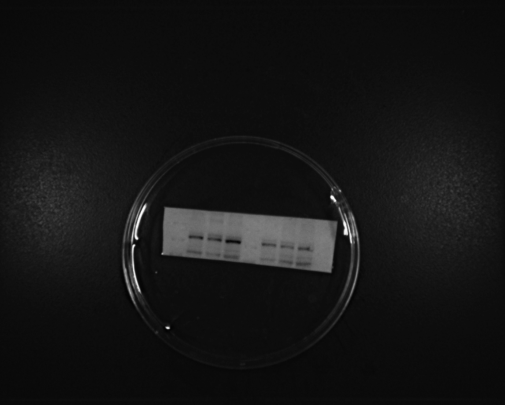
**STIR1**


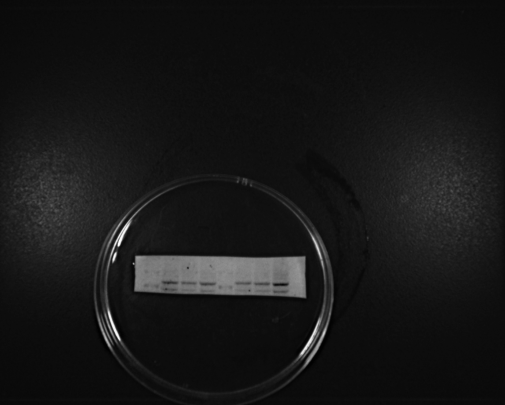
**PGC-1a**


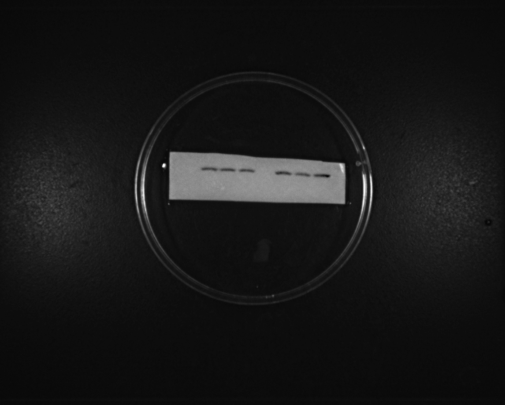
**β-actin**

Supplement: Supplementary file 1 [file DataSheet1.docx]
